# Breeding on the leading edge of a northward range expansion: differences in morphology and the stress response in the arctic Gambel’s white-crowned sparrow

**DOI:** 10.1007/s00442-015-3447-7

**Published:** 2015-10-01

**Authors:** Jesse S. Krause, Helen E. Chmura, Jonathan H. Pérez, Lisa N. Quach, Ashley Asmus, Karen R. Word, Michaela A. McGuigan, Shannan K. Sweet, Simone L. Meddle, Laura Gough, Natalie Boelman, John C. Wingfield

**Affiliations:** Department of Neurobiology, Physiology and Behavior, University of California Davis, One Shields Avenue, Davis, CA 95616 USA; Department of Biology, University of Texas at Arlington, Arlington, TX 76019 USA; Roslin Institute, Royal (Dick) School of Veterinary Studies, University of Edinburgh, Easter Bush, Midlothian, EH25 9RG Scotland, UK; Department of Earth and Environmental Sciences, Lamont-Doherty Earth Observatory of Columbia University, Palisades, NY 10964 USA

**Keywords:** Adrenocortical, Corticosterone, Hypothalamic–pituitary–adrenal axis, Glucocorticoids, Morphometrics, Climate change, Allostatic load

## Abstract

Individuals at the forefront of a range shift are likely to exhibit phenotypic traits that distinguish them from the population breeding within the historic range. Recent studies have examined morphological, physiological and behavioral phenotypes of individuals at the edge of their range. Several studies have found differences in the hypothalamic–pituitary–adrenal (HPA) axis activity in response to acute restraint stress in individuals at the range limits. HPA axis activation leads to elevations in glucocorticoids that regulate physiology and behavior. Here we compare the hormonal profiles and morphometrics from Gambel’s white-crowned sparrows (*Zonotrichia leucophrys gambelii*) breeding at the northern limit of the population’s range to those birds breeding within the historic population range. Birds breeding at the northern limit experienced a harsher environment with colder temperatures; however, we found no differences in arthropod prey biomass between the northern limit and more southern (historic) sites. Males at the northern limit had higher body condition scores (mass corrected for body size) compared to individuals within the historic range, but no differences were found in beak and tarsus lengths, wing chord, muscle profile or fat stores. In males during the pre-parental stage, before breeding commenced, HPA axis activity was elevated in birds at the northern limit of the range, but no differences were found during the parental or molt stages. Females showed no differences in HPA axis activity during the parental stage. This study suggests that “pioneering” individuals at the limits of their breeding range exhibit physiology and morphology that are distinct from individuals within the historic range.

**Electronic supplementary material:**

The online version of this article (doi:10.1007/s00442-015-3447-7) contains supplementary material, which is available to authorized users.

## Introduction

The geographical range of a species is determined by the ecological niche where evolution has selected the traits that maximize fitness (MacArthur 1972). Species ranges are often highly plastic, shifting or expanding to match spatial shifts of their niche (Sexton et al. 2009). As an individual moves to the range margins, the specialized traits may no longer be optimal for the environment that is encountered because habitat quality has declined. (Sexton et al. 2009). Changes in species’ geographic range are one of the most commonly reported responses to climate change. A meta-analysis of 1700 plant and animal species indicates that ranges are shifting at a rate of 6.1 km per decade towards the poles (Parmesan and Yohe 2003). Northward range expansion may expose individuals to more spatially disparate habitats, shifts in phenology and abundance of prey items, novel parasites and predators, and to thermal challenges that have not previously been experienced.

Individuals at the leading edge of a range expansion may exhibit distinct behavioral, morphological, and physiological traits that enhance fitness in a challenging environment. There have been many behavioral traits associated with a vertebrate’s ability to colonize a new area that include aggression, sociability, boldness, and exploration (Cote et al. 2010). Morphological characteristics, such as body size (Bowler and Benton 2005), are also commonly reported as observed changes in range expanding individuals, and evidence exists for both larger and smaller individuals moving into new areas (Bowler and Benton 2005). In addition, circulating levels of glucocorticoids are commonly measured because these hormones regulate physiology and behavior and are thought to help these individuals in their novel environment (Addis et al. 2011; Dunlap and Wingfield 1995; Liebl and Martin 2012).

Modulation of glucocorticoid secretion by the hypothalamic–pituitary–adrenal (HPA) axis may be particularly important in vertebrate populations undergoing a range expansion because it is a highly plastic trait that is profoundly influenced by the environment. Stressors in the environment, such as storms, predation attempts, food shortages, and territorial disputes induce increases in glucocorticoids that regulate changes in physiology and behavior to enhance fitness (Sapolsky et al. 2000; Wingfield and Sapolsky 2003). For birds, the main glucocorticoid, corticosterone, is important for energetics (Astheimer et al. 2000; Landys et al. 2004), foraging (Angelier et al. 2008; Astheimer et al. 1992), dispersal (Silverin 1997) and escape behavior (Breuner and Hahn 2003). Basal levels of corticosterone are important for regulating metabolic processes (Sapolsky et al. 2000). Acute activation of the HPA axis, in response to stress, is beneficial to the organism, but chronic activation can have deleterious effects (Sapolsky et al. 2000; Wingfield and Sapolsky 2003). Since glucocorticoids are an important mediator of life history trade-offs, either under- or over-production of these steroids could have significant fitness consequences (i.e., the decision to abandon a nest) (Angelier and Wingfield 2013).

Existing evidence indicates that birds breeding at the edge of a population’s range show increased HPA activity, as well as higher variance in activity in response to acute restraint stress, compared to individuals within the historic range (Addis et al. 2011; Liebl and Martin 2012; Walker et al. 2015; Wingfield et al. 1995a). Range expansion into a new territory with novel characteristics such as harsher climate, interactions with novel species, altered habitat characteristics such as plant species richness, abundance, and density; and altered food availability and quality; may make elevated HPA axis activity beneficial for coping with these environmental stressors (Wingfield et al. 2015).

The Arctic is changing rapidly in response to climate change and provides a unique system to investigate characteristics of individuals at the leading edge of a range expansion. The expansion of deciduous woody shrubs, mainly willows (*Salix* spp.), alders (*Alnus* spp.), and birch (*Betula* spp.) (Sturm et al. 2001; Tape et al. 2006), has transformed the landscape enabling colonization by higher trophic level species previously limited by habitat quality. One colonist of this new habitat is a migratory bird, the Gambel’s white-crowned sparrow (*Zonotrichia leucophrys gambelii*), which winters in the North American Southwest and migrates to its breeding grounds that span a large geographical area extending from Southern to Northern Canada and Alaska (Chilton et al. 1995). Based on observations by our laboratory over the last 27 years, Gambel’s white-crowned sparrows have tracked the northward expansion of woody shrubs, north of the Brooks Mountain Range in Alaska, and thus have expanded their breeding distribution on the North Slope towards the Arctic Ocean (Wingfield, personal observation). This is corroborated by Breeding Bird Survey data from the US Geological Survey that indicate that the number of birds breeding north of the Brooks Range has increased in the past 8 years (Sauer et al. 2014 ) (Supplemental Fig. 1).

To date, little is known about any adaptive traits that will be beneficial to arctic breeding songbirds colonizing new areas. Here we established whether individuals at the forefront of the range exhibit physiological and morphological characteristics that are distinct from those of individuals in the historic range as a consequence of differing environments. Potential differences in physiology and morphology could be a consequence of either phenotypic plasticity or genetic adaptation. We hypothesized: (1) that birds breeding at the northern limit would experience harsher environments characterized by colder temperatures, higher wind speeds, and lower arthropod biomass (an indicator of food availability). As a consequence of the harsher environment, we hypothesized: (2) that HPA activity in response to acute restraint stress, as well as morphological measures, would be distinct between birds at the edge of the population’s range compared to birds within the historic range, and that these parameters differ among stages of breeding. We predicted that birds at the range limits would show greater basal and stress-induced levels of glucocorticoids and enhanced body condition that include increased mass, fat stores and flight muscle profile when compared to birds within the historic range for each stage of breeding. At both the range limit and historic range, we predicted that HPA axis activity would be highest during the pre-parental stage when conditions are harshest, and that birds would have reduced fat stores, muscle and mass compared to the parental and molt stages.

## Materials and methods

### Study sites and species

**Fig. 1:**
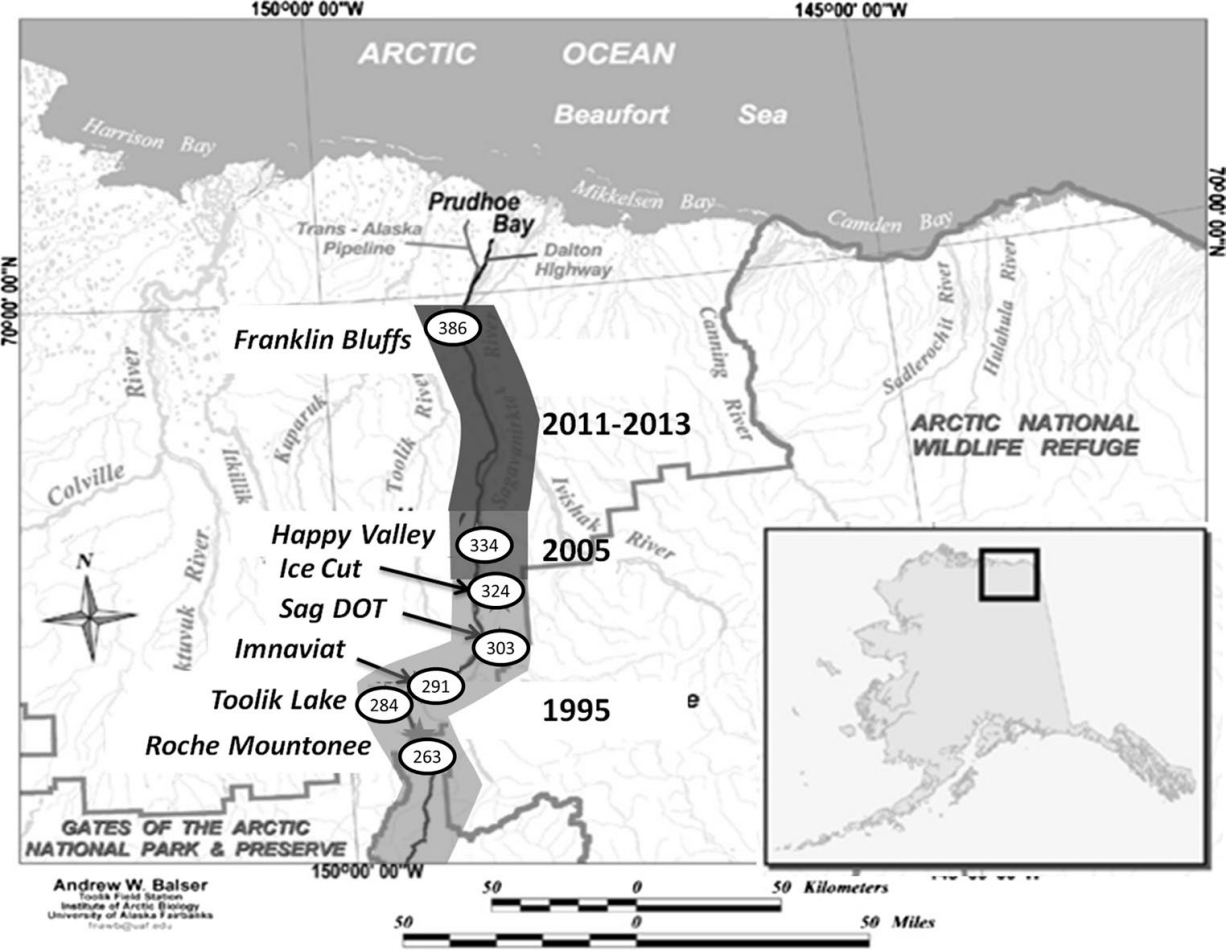
Breeding range map for the Gambel’s white-crowned sparrow along James Dalton Highway, Alaska. Site locations are listed as mile point numbers reflecting distances from the start of the James Dalton Highway in the south and terminating at mile point (MP) 414 in Prudhoe Bay. In the early 1990s, the population’s range ended at Ice Cut at MP 324 (*light-gray* *shading*). In 2005, males were found at Happy Valley at MP 334 (*medium-gray shading*). By 2011, breeding populations were found as far north as Franklin Bluffs at MP 386 (*dark-gray* *shading*). This evidence suggests a northward range expansion

Our field operation was based at Toolik Lake Field Station at mile point (MP) 284 on the James Dalton highway in the foothills of the Brooks Mountain Range on the North Slope of Alaska, USA (69°38′N, 149°36′W). The James Dalton Highway starts at MP 1 in the south and terminates at 414 in the north at Deadhorse; this creates an ideal latitudinal transect for sample collection. Climate, bird, and arthropod data were collected at the range-limit site, MP 386 (Franklin Bluffs; 69°50′N, 148°45′W), as well as at the four historic range sites, MP 303 (Sagavanirktok Department of Transportation; 68°45′N, 148°53′W), MP 291 (Imnavait Creek; 68°37′N, 149°18′W), MP 284 (Toolik Lake Field Station), and MP 265 (Roche Mountonee; 68°22′N, 149°19′W) (Fig. 1). The distance from the Toolik Lake Research Station to the Franklin Bluffs is 102 miles (164 km) by road while the geographical distance is 86 miles (122 km).

Breeding Bird Survey data indicate that the number of Gambel’s white-crowned sparrows encountered along a point-count transect greatly diminishes north of the Brooks Range in Alaska, and although these numbers seem to fluctuate between years, they are currently increasing (Sauer et al. 2014) (Supplemental Fig. 1). The decline in bird numbers from south of the Brooks Range to the northern limit of the range can be attributed to reductions in suitable habitat as shrubs become patchier and more disparate. In 1987, the breeding range extended north to MP 303 on the James Dalton Highway (Fig. 1). In 2005, birds were present at MP 334 (Happy Valley; 69°09′N, 148°49′W) although no evidence of breeding was found. However in 2011, birds were found breeding further north around MP 386 and two non-breeding males were found 16 km further north at MP 396 (69°58′N, 148°42′W). The edge of the population’s range has moved a geographical distance of 75 miles (120 km) north in 24 years with a population estimated to be between 24 and 40 birds, depending on the sampling year, which have successfully reproduced (Wingfield, unpublished data). In the present study, birds inhabiting areas colonized after 2005 (MP 386) are termed “range limit” and birds found in existing range (MP 266–303) are termed “historic range”.

Gambel’s white-crowned sparrow is a socially monogamous, long-distance migrant that raises a single brood during the short arctic summer (Chilton et al. 1995). Birds typically arrive on their breeding grounds in mid-May when snow cover is abundant and they remain in small flocks until enough snow melts to permit territory establishment (Wingfield et al. 2004). Females are responsible for nest construction which can last from 1 to 3 days (Oakeson 1954). A typical clutch consists of four to five eggs with one egg laid per day (Norment 1992; Oakeson 1954). The brood patch may develop up to 5 days prior to lay but typically becomes fully edematous at the onset of incubation (Bailey 1952). Mean clutch initiation dates within the historic range were 1 June in 2011 and 3 June in 2012 (Wingfield et al., unpublished data). The eggs are incubated for approximately 12 days until hatch and nestlings remain in the nest approximately 9 days until fledge (Norment 1992). Fledglings are fed by the parents until they are fully independent. Molt in adults typically starts after nestlings fledge, at the beginning of July and last until mid- to late August (Morton et al. 1969). Fall departure from the North Slope of Alaska can occur from late August to early September (Ramenofsky and Chmura, unpublished data).

Gambel’s white-crowned sparrows prefer to breed in a habitat that contains shrubs, grass, and a nearby water source (Boelman et al. 2015; Chilton et al. 1995). Nests are typically found either in *Betula* or *Salix* species ranging in height from 20 cm to ~1 m (Boelman et al. 2015). Thus a certain threshold of shrub density, complexity, and height appears to be a requisite for suitable nesting habitat (Boelman et al. 2015; Norment 1993; Oakeson 1954). All blood samples were collected from the birds in accordance with University of California Davis Institutional Animal Care and Use Committee approval under protocol 17812.

### Climatic variables

Weather data were collected by meteorological stations at one range-limit site (Kane 2013) and four historic range sites (Environmental Data Center 2013). Our analyses focused on minimum temperatures, wind speed, and wind chill as indicators of environmental harshness at three different stages of the breeding season which included pre-parental, parental and molt. Wind chill was calculated because for a set temperature, increases in wind velocity enhance cooling rates through convective heat loss and as a result lower the apparent temperature. Wind chill was calculated with the equation *T*_WC_ = 13.12 + 0.6215*T*_a_ − 11.37*V*^0.16^ + 0.3965*T*_a_*V*^0.16^ where *T*_WC_ is wind chill, *T*_a_ is ambient temperature and *V* is wind velocity (Osczevski 1995). Snow cover was less than 20 % at the onset of the study and as a result was not included in our analyses.

### Arthropod biomass

Canopy-dwelling arthropod biomass was measured weekly during the 2012 field season using a standard sweep net protocol to capture arthropods (Boelman et al. 2015). At each site, an area characterized by dwarf deciduous shrub tundra was designated for sampling and an iron rod was driven into the ground to serve as a central point for a 100-m transect. After generating a random bearing for the transect, by spinning a pen, a total of ten sweep net samples were collected at 10-m intervals along the transect. Individual samples were collected by making 20 horizontal passes along the vegetation. Individual sample contents were placed into a plastic bag containing a 1-cm^2^ piece of Hot Shot No-pest strip to euthanize the arthropods, and then put in a −20 °C freezer upon return to the laboratory. Arthropods were then separated from plant material, placed into Petri dishes, dried for 24 h at 40 °C, and then weighed to the nearest milligram.

### Bird morphometrics and HPA axis activity

Birds were sampled at three distinct stages across the breeding season in 2011 and 2012. The pre-parental stage lasted from arrival on territories, as determined by the return of color-banded individuals to known territories, to average clutch initiation date and was classified using mean clutch initiation dates from historic sites in conjunction with brood patch development from both historic and range-limit sites. During the pre-parental stage, developing brood patches were recorded in females at both range-limit and historic sites suggesting that breeding phenology was similar between the two sites. In addition, no edematous brood patches were recorded suggesting that incubation had not commenced (see Bailey 1952 for description). The parental phase included incubation and feeding young until molt was initiated, at which point birds were classified as no longer in breeding condition. Samples for the pre-parental stage, parental, and molt were collected on 20 May to 4 June, 23 June to 11 July, and 7–28 July, respectively.

A standard capture restraint protocol was used to measure HPA axis activity (Wingfield et al. 1992). A total of 57 birds were captured using Japanese mist nets in conjunction with song playback. Following venipuncture of the wing vein with a 26-gauge needle, a baseline blood sample was collected into 75-µL microhematocrit tubes within 3 min of capture. Between blood sampling at 10-, 30- and 60-min post-capture the bird was placed into an opaque cloth bag. Samples from females were also collected but we only had a sufficient number to include birds from the parental stage. After blood collection, each bird was banded with an aluminum US Geological Survey band along with a unique combination of color bands for identification in the field. Prior to release, morphometrics of wing chord, tarsus length, and beak length (nares to tip) were measured using calipers (±0.1 mm); and mass was measured using a Pesola spring scale (±0.1 g). Composite fat stores (furcular and abdominal) on a scale from 0 (lean) to 5 (fat) (Wingfield and Farner 1978) and muscle profile on a scale from 0 to 3 (Bairlein and Simons 1995) were also recorded. The blood was kept chilled for 6–12 h until processing. Blood samples were centrifuged at 10,000 r.p.m. for 5 min to separate the plasma. Plasma was aspirated with a fixed-needle Hamilton syringe, placed into a microcentrifuge tube, and stored at −80 °C. At the end of the field season, all samples were transported frozen on dry ice to the University of California, Davis.

### Radioimmunoassay for corticosterone

A radioimmunoassay was used to quantify plasma concentrations of corticosterone (Wingfield et al. 1992). Tritiated corticosterone was purchased from Perkin Elmer (NET399250UC) and corticosterone antibody from Esoterix (B3-163). Samples were assayed in duplicate and counted for 10 min or to within 2 % accuracy on a Beckman LS 6500 counter. Mean recoveries were 84.57 %. Inter- and intra-assay variations were 15.4 and 10.7 %, respectively.

### Statistical analyses

Statistical analyses were performed using JMP 11 (1989-2007; SAS Institute, Cary, NC) and in R using package Pracma [Borchers (2015), practical numeric math functions (Pracma); R Package version 1.6.4. http://CRAN.R-project.org/package=pracma]. Climatic variables including minimum temperature, wind chill and mean wind speed were analyzed using a two-way factorial ANOVA to investigate the effects of breeding stage (pre-parental, parental and molt) and range (limit and historic). Post hoc tests were performed using Tukey’s honest significant difference (HSD) test. Sweep net data were analyzed with a repeated-measures ANOVA to investigate the effects of range and sampling week on arthropod biomass.

Tarsus, beak, and wing chord were compared using a *t-*test to investigate morphometric differences between historic and range-limit birds. In order to correct for body size effects on mass, we conducted a principal component (PC) analysis using beak and tarsus lengths and wing chord to create a structural size index (PC1). The residuals from the regression of mass against the PC1 were used to create a corrected body condition index. Morphometrics including fat stores, body index, and muscle profile were analyzed using a two-way factorial ANOVA including the main effects of range and breeding stage and their interaction. Tukey’s HSD post hoc analyses were used following a significant ANOVA to determine differences among groups.

Hormonal data were analyzed using two statistical approaches following log transformation. All observations within a life history stage were from unique individuals while at the range-limit site four birds were sampled at two different life history stages. Multivariate repeated-measures ANOVA was used to evaluate the effects of range (historic vs. range limit), stress response (effect of acute restraint handling), and their interaction on corticosterone at baseline, 10, 30 and 60 min post-sample at each breeding stage. Data were checked for sphericity, and when not met, the Greenhouse–Geisser correction was used. Differences in integrated corticosterone measurements were tested using a general linear model with the effects of range, breeding stage and their interaction. Post hoc analyses were performed using a Student’s *t*-test to compare integrated corticosterone within a breeding stage. Integrated corticosterone was calculated by using the trapezoidal rule between time points and subtracting baseline. Data are reported as the mean ± SEM.

## Results

### Climatic variables

**Fig. 2:**
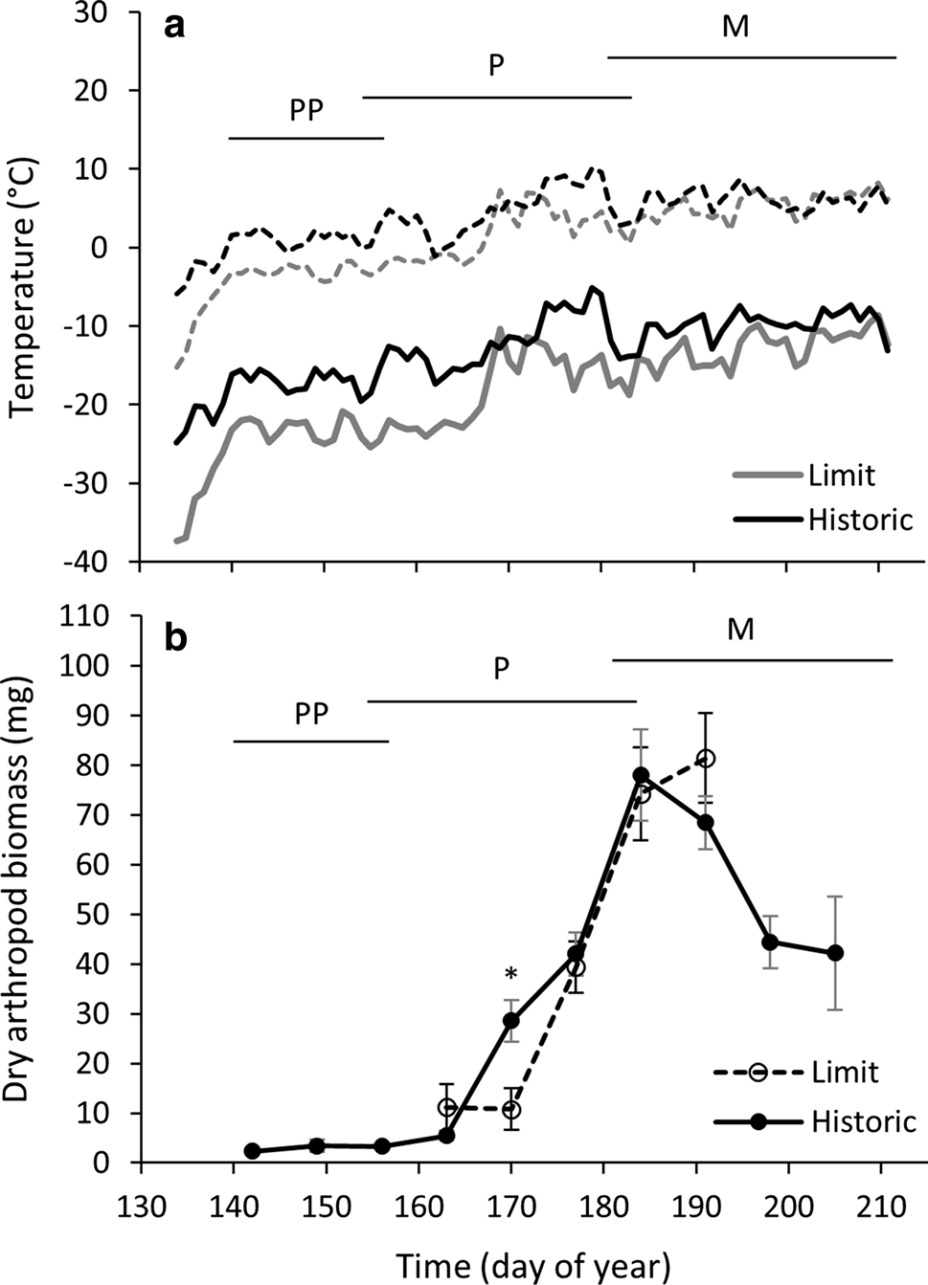
Average daily **a** minimum ambient temperatures, wind chill temperatures and weekly **b** arthropod dry biomass, collected using sweep net sampling, during the breeding season within the historic range (*Historic*) and at the range limit (*Limit*). The timing of pre-parental (*PP*), parental (*P*), and molt (*M*) stages are indicated by the *horizontal lines* at the* top* of* each graph*. Minimum ambient temperatures were colder at the range limit (*grey dotted line*) compared to the historic range (*black dotted line*) during the pre-parental and parental stages but not during the molt stage. Due to higher wind speeds at the range limit, colder wind chill temperatures were experienced at the range limit (*grey solid line*) than the historic range (*black solid line*) at all stages. Arthropod biomass was significantly different between the range limit and historic range at only one time point (18 June 2012, i.e., day of year 170). Values are presented as mean ± SEM. *P < 0.05

Temperatures were different across stages of breeding (*F*_2,2_ = 190.66, *P* < 0.01; Fig. 2a). There was a main effect of range on minimum temperature (*F*_1,1_ = 48.15, *P* < 0.01) and mean wind speed (*F*_1,1_ = 198.34, *P* < 0.01). The interaction of range and breeding stage was significant for minimum temperature (*F*_2,2_ = 10.11, *P* < 0.01) and mean wind speed (*F*_2,2_ = 5.60, *P* = 0.003). Temperatures were colder at range limit than historic range during the pre-parental (*t* = 5.98, *P* < 0.001) and parental (*t* = 3.72, *P* < 0.001) stages but not during molt (*t* = 1.90, *P* < 0.06). Tukey’s post hoc analyses indicated that mean wind speed was higher at the range limit at each stage (Supplemental Fig. 2). When investigating effects of wind chill, the magnitude of the difference in temperatures between historic range and range limit were significantly increased (*F*_1,1_ = 132.38, *P* < 0.001) at the pre-parental (*t* = 23.01, *P* < 0.01), parental (*t* = 13.85, *P* < 0.01) and molt (*t* = 13.96, *P* < 0.01; Fig. 2a) stages.

### Arthropod biomass

Dry arthropod biomass significantly increased as the season progressed for both historic and range-limit sites (*F*_4,45_ = 36.11, *P* < 0.01; Fig. 2b). However, biomass was not different between the two locations (*F*_4,48_ = 0.21, *P* = 0.64). The interaction between range and sampling week was significant (*F*_4,45_ = 58.69, *P* = 0.05). Tukey’s post hoc analyses indicated that biomass was higher at the historic site compared to the range-limit site during the week of 18 June 2012 (day of year 170).

### Bird morphometrics

#### Body size

Measures of wing chord (male, *t* = 1.05, *P* = 0.26; female, *t* = 0.94, *P* = 0.35), tarsus (male, *t* = 1.34, *P* = 0.18; female, *t* = 0.52, *P* = 0.60), and beak length (male, *t* = 0.17, *P* = 0.17; female, *t* = 0.19, *P* = 0.84) were not different between historic and range-limit sites.

#### Body condition

**Fig. 3:**
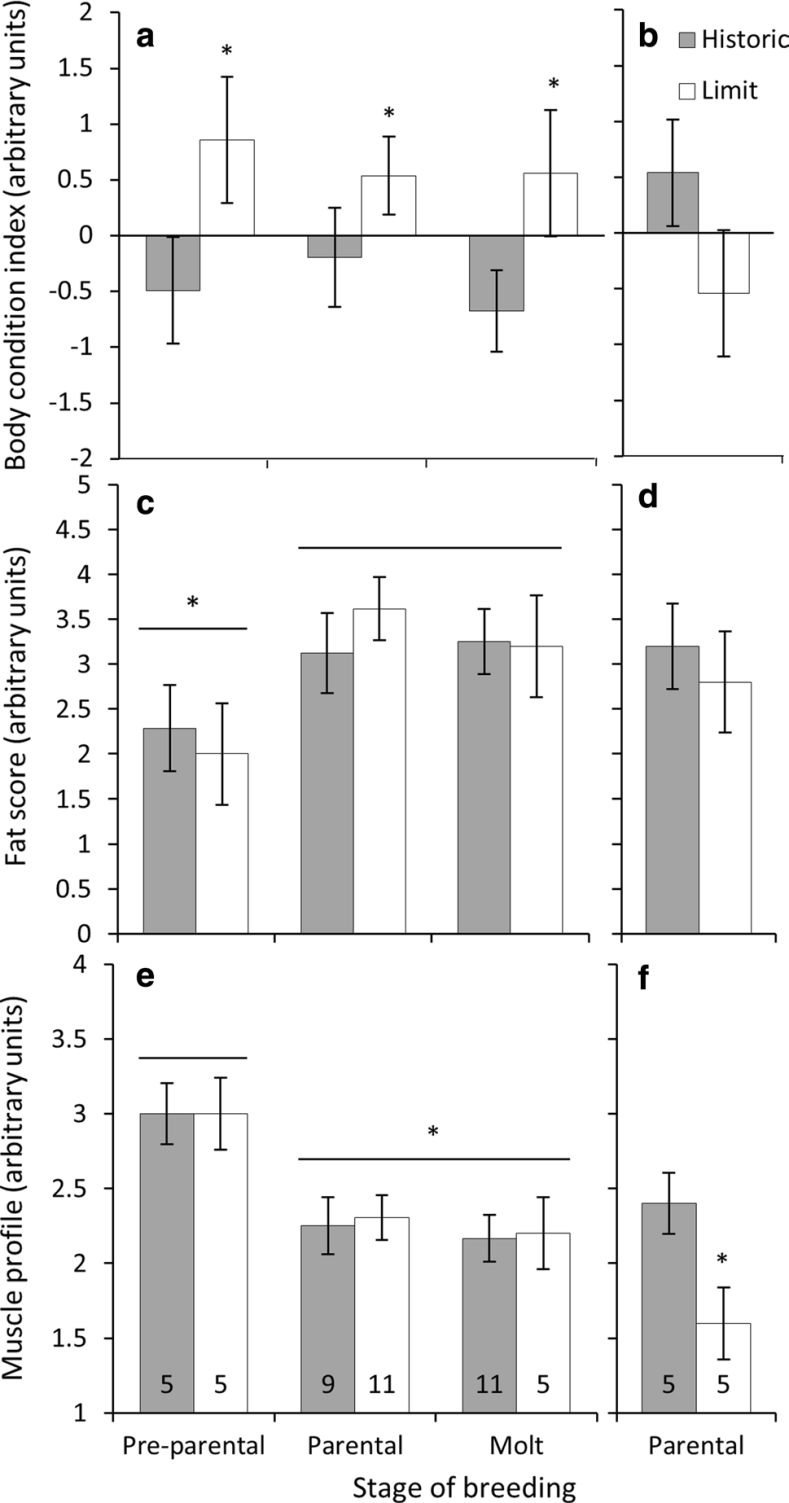
The effect of geographical range location on body condition index, total fat stores, and muscle profile in Gambel’s white-crowned sparrow **a**, **c**, **e** males during the pre-parental, parental and molt stages and in **b**, **d**, **f** females during the parental stage. Significant differences between range limit (*Limit*) and historic range (*Historic*) were observed for body condition in **a** males, and muscle profile in **f** females. In males, regardless of range, **c** fat stores were smaller and **e** muscle profiles were larger during the pre-parental stage compared to all other stages. Body index scores were generated by performing a principal components (PC) analysis for tarsus, wing, and beak measurements. The PC1 score was saved, regressed against body mass, and then the residuals were used for range comparison. Values are presented as mean ± SEM. **e**, **f** *Numbers within bars* indicate sample sizes for each group. *P < 0.05

Males at the range-limit site had a higher body condition index (*F*_1,44_ = 5.62, *P* = 0.02; Fig. 3a). Male body condition index did not change across the breeding season (*F*_2,44_ = 0.11, *P* = 0.89) and the interaction of breeding stage and range was not significant (*F*_2,44_ = 0.18, *P* = 0.83). For females the body index during the parental stage was not different between range-limit and historic sites (*t* = −1.21, *P* = 0.26; Fig. 3b).

#### Fat stores

In males, fat stores were different across breeding stages (*F*_2,44_ = 3.74, *P* = 0.03; Fig. 3c). Males stored less fat during the pre-parental compared to the parental (*t* = 5.84, *P* < 0.001) and molt (*t* = 4.63, *P* < 0.001) stages. In males, the effect of range (*F*_1,44_ = 0.01, *P* = 0.89) and the interaction of range and breeding stage (*F*_2,44_ = 0.39, *P* = 0.67) were not significant. Female fat stores were not different between historic range and range limit (*t* = −0.45, *P* = 0.66; Fig. 3d).

#### Muscle profile

Muscle profiles in males varied across breeding stages (*F*_2,44_ = 8.81, *P* < 0.01; Fig. 3e). In males at both the range limit and historic range, muscle profiles were larger during the pre-parental compared to parental (*t* = 7.26, *P* < 0.001) and molt (*t* = 6.76, *P* < 0.001) stages. Muscle profiles in males were not different between birds at the historic or range-limit sites (*F*_1,44_ = 0.03, *P* = 0.85). The interaction between stage of breeding and range in males was not significant (*F*_2,44_ = 0.01, *P* = 0.98). The muscle profile for females was smaller at the range limit (*t* = −2.30, *P* = 0.047; Fig. 3f).

#### HPA axis activity: corticosterone measurement

Male basal corticosterone values showed an interaction between range and breeding stage (*F*_5,40_ = 5.18, *P* = 0.01). Basal corticosterone values were higher in males at the range limit during the pre-parental stage only (*t* = 5.48, *P* = 0.05).

**Fig. 4:**
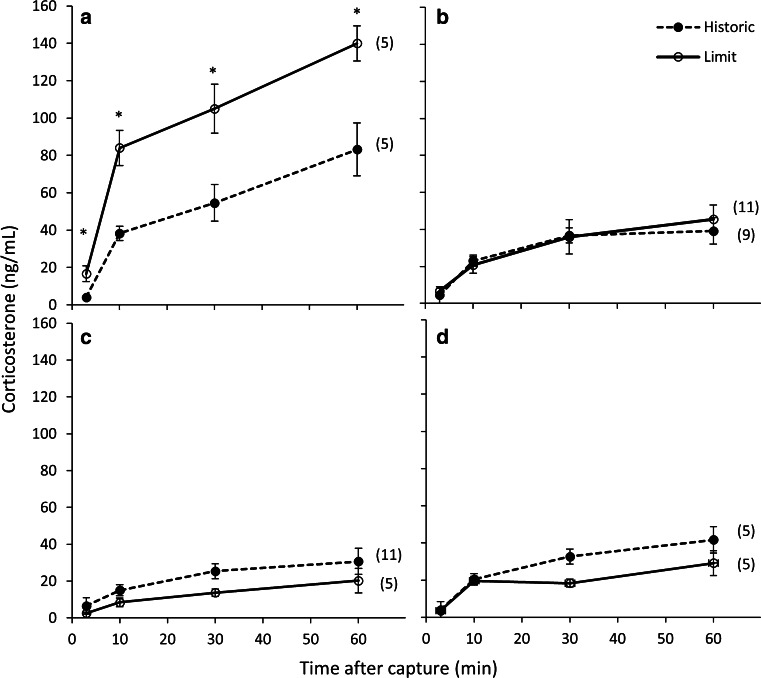
The effect of geographical range location on corticosterone levels in response to standard restraint stress in Gambel’s white-crowned sparrow males during **a** pre-parental, **b** parental, and **c** molt stages and in females during **d** the parental stage. During the pre-parental stage, the corticosterone levels were higher at each time point in males at the range limit (*Limit*) compared to the historic range (*Historic*), while no differences were observed for males or females at any other stage. *Numbers in parentheses* indicate sample size for each group. Values presented as mean ± SEM. *P < 0.05

For males, corticosterone levels increased from basal levels in response to capture and restraint stress during pre-parental (*F*_3,6_ = 49.74, *P* < 0.01; Fig. 4a), parental (*F*_3,16_ = 39.62, *P* < 0.01; Fig. 4b) and molt stages (*F*_3,13_ = 11.17, *P* < 0.01; Fig. 4c). During the pre-parental stage, the stress response was elevated in birds at the range limits compared to the historic range (*F*_1,8_ = 13.29, *P* < 0.01) but no differences were found during the parental (*F*_1,18_ = 0.002, *P* = 0.96) or molt stages (*F*_1,15_ = 0.88, *P* = 0.36). During the pre-parental stage corticosterone levels were higher in the range limit birds at 10 (*t* = 2.38, *P* = 0.03), 30 (*t* = 2.27, *P* = 0.04) and 60 (*t* = 3.14, *P* = 0.009) min. The interactions between stress and range were not significant during pre-parental (*F*_3,6_ = 1.85, *P* = 0.20), parental (*F*_3,25_ = 0.51, *P* = 0.60), and molt stages (*F*_3,13_ = 0.07, *P* = 0.97).

In males, integrated corticosterone levels were highest during the pre-parental stage compared to parental and molt stages (*F*_6,48_ = 40.91, *P* = 0.001). The interaction of breeding stage and range was significant (*F*_2,48_ = 6.47, *P* = 0.003) so integrated corticosterone levels were compared within each stage. Integrated corticosterone was higher in birds at the range limit than the historic range during the pre-parental stage (*t* = 3.97, *P* = 0.004) but no differences were found during the parental (*t* = 0.02, *P* = 0.98) or molt (*t* = 1.65, *P* = 0.11) stages.

For females, corticosterone levels increased from basal levels in response to capture and restraint stress (*F*_3,6_ = 32.62, *P* < 0.01; Fig. 4d). The corticosterone levels in response to acute restraint stress were not different between the historic and range-limit sites (*F*_1,8_ = 4.31, *P* = 0.07). Integrated corticosterone was also not different between historic and range-limit sites (*t* = −2.07, *P* = 0.07).

## Discussion

The range of Gambel’s white-crowned sparrows has expanded northwards over the past 30 years and we suggest that this has been facilitated by an expansion of deciduous woody shrubs leading to an increase in available breeding habitat. Relative shrub cover has increased 63 % over the past 50 years along the Sagavanirktok River which is the second largest drainage on the North Slope and encompasses our study sites (Tape et al. 2006). As a consequence of the range expansion, compared to more southern sites, birds breeding at the northern edge of the range experienced a harsher environment that featured colder temperatures, especially when accounting for wind chill. This is not surprising, since it is known that spring and summer temperatures are typically warmer in the foothills of the Brooks Range and decline towards the Arctic Ocean (Kade et al. 2005; Nelson et al. 1997). Sweep net arthropod biomass was significantly different at only one time point, which may suggest a slightly later emergence of arthropods at the range limit; however, no differences were found later in the season. We also cannot discount other potential differences in food resources such as the abundance of berries. This study investigated the adjustments in morphology and physiology necessary for range expansion and colonization of a harsh environment.

Male sparrows breeding at the limits of their range possessed significantly higher body condition indices. This pattern could be caused either by differences in the birds that initially arrive at the range limit or by differences in birds that successfully gain territories and breed at northern sites; we do not have data that will allow us to conclusively disentangle these competing explanations. However, existing evidence suggests that these are phenotypic traits that are associated with individuals that are responsible for dispersing into new areas. Studies in naked mole rats (*Heterocephalus glaber*), side-blotched lizards (*Uta stansburiana*), and Belding’s ground squirrels (*Urocitellus beldingi*) have shown that dispersing individuals often have a higher body mass than non-dispersers (Sinervo et al. 2006; Holekamp 1986; O’Riain et al. 1996). Additionally, increased mass may be important for surviving in a harsher environment by increasing thermal inertia according to Bergmann’s rule (Kendeigh 1969). Tarsus, wing chord and beak length were not different between the two sites suggesting that there are no structural differences in size between the two populations. This evidence, in conjunction with our current findings, suggests that heavier birds for their body size and, therefore, perhaps higher quality Gambel’s white-crowned sparrows, may be responsible for extending their population’s range. Interestingly, the difference in mass could not be attributed to larger muscle profiles or fat stores in the birds at the range limit. This would suggest that other components of the bird’s morphology are generally larger or heavier and were not elucidated by our measurements.

We found basal levels of corticosterone to be elevated in male birds at the range limit during the pre-parental stage. Our findings are similar to results obtained from Puget Sound white-crowned sparrows (*Zonotrichia leucophrys pugetensis*) breeding in novel high-altitude sites. Populations of *Z. leucophrys pugetensis* have significantly elevated levels of basal corticosterone with increasing altitude, which the authors attributed to harsher, colder and less familiar conditions than previously encountered by this sub-species breeding at lower altitudes (Addis et al. 2011). The *Z. leucophrys pugetensis* study findings are, however, contradicted by those from studies of an introduced population of house sparrows (*Passer domesticus*) undergoing range expansion in Africa and in female dark-eyed juncos (*Junco hyemalis*) invading the urban areas from surrounding montane areas in San Diego, California in which the basal corticosterone levels did not differ between birds on the edge of the population’s range compared to birds breeding within the historic range (Atwell et al. 2012; Liebl and Martin 2012). However, in both of these studies it is unlikely that the birds were faced with thermally challenging temperatures.

One potential explanation for elevated basal corticosterone levels in birds at the northern limit of the range is that food resources were limited during the early season, forcing birds to draw on energy stores to meet increased metabolic demands associated with a harsher environment. Corticosterone has an effect on many catabolic processes such as liberation of lipids from adipose tissues and the breakdown of protein for glucose production (Sapolsky et al. 2000). In birds, corticosterone implants lead to declines in muscle profile and fat stores (Astheimer et al. 2000). However, in males the inability to meet energetic demands is not supported by our fat or muscle data as we did not detect differences in fat stores or muscle profiles between individuals at the range limit and southern sites during any stage of breeding. This does not disprove the assumption that increased corticosterone levels are contributing to the regulation of metabolism, but instead suggests that any catabolic effects of corticosterone must have been offset by increased food intake.

An alternative explanation for elevated basal corticosterone levels in birds at the northern limit of the range is that food resources were limited during the early season, but that rather than drawing on energy reserves the birds were able to increase foraging effort to meet this demand. Corticosterone implants have been shown to increase foraging (Astheimer et al. 1992; Angelier et al. 2007) and increase activity range (Breuner and Hahn 2003). Our sweep net sampling showed that arthropod biomass was comparable at the two sites, other than the week of 18 June 2012, suggesting that canopy and airborne arthropods were equally abundant across the range. However, during the pre-parental stage, arthropod dry biomass was low and birds may have been reliant upon other food sources such as berries (*Vaccinium uliginosum*, *Empetrum nigrum*, *Vaccinium vitis*-*idaea*, and *Arctous rubra*) and catkins (*Betula* spp. and *Salix* spp.). Plant community surveys in the area surrounding Franklin Bluffs, which is dominated by wet non-tussock tundra, indicate that berry-producing species are uncommon and thus would be a limited resource (Kade et al. 2005). This is contrasted by the abundance of berry-producing species found in moist acidic tussock tundra within the historic range (Walker et al. 1994). If food resources are different between the sites during the pre-parental period, then the disparity in observed corticosterone might be a requisite of northern birds to increase foraging effort and activity range to maintain body weight. The difference in basal corticosterone was absent during the parental or molt stages in both sexes despite the colder ambient temperatures during the parental stage. However, during these later breeding stages, arthropod dry biomass is increasing and temperatures remain in the vicinity of the thermal neutral zone. Therefore, elevated levels of corticosterone are no longer necessary to increase foraging effort, activity range, nor catabolic processes in order to meet energetic demands.

HPA axis activity and release of corticosterone in response to capture restraint stress was significantly higher in males breeding at the range limit compared to individuals at historic sites, but only during the pre-parental stage. This is consistent with studies in other bird species such as house sparrows (Liebl and Martin 2012), Puget Sound white-crowned sparrows (Addis et al. 2011), Lapland longspurs (*Calcarius lapponicus*), snow buntings (*Plectrophenax nivalis*) (Walker et al. 2015), bush warblers (*Cettia diphone*) (Wingfield et al. 1995a) but also in western fence lizards (*Sceloporus occidentalis)* which showed that individuals at the edge of their range have an elevated HPA axis activity in response to acute restraint stress. In addition, a significant relationship exists between latitude and HPA activity in birds (Jessop et al. 2013). The activity of the HPA axis is highly plastic and is influenced by environmental harshness in order to promote survival (Boonstra 2004; Wingfield 2008). The influence of environmental factors is exemplified in captive and field studies, when in the absence of environmental and social stimuli, HPA axis activity is greatly attenuated (Romero and Wingfield 1999) while it is up-regulated in response to increased stimuli such as storms (Astheimer et al. 1995; Rogers et al. 1993; Wingfield et al. 1983). Corticosterone has been proposed to be a key mediator of trade-offs between the current life history stage and the emergency life history stage (Wingfield et al. 1995b). Entry into the emergency life history stage results in the abandonment of the current life history stage and redirection of resources to self-preservation which can have detrimental consequences on reproductive output. Elevation in HPA activity at the range limit of a population, and the rapid rise of corticosterone levels in response to stress, may be critical for determining the success of the individual in a novel environment as it promotes rapid changes in behavior and physiology. The fact that not all individuals within our study population demonstrated the same HPA axis activity, suggests that there are benefits associated with elevated corticosterone that enhance self-preservation but may also be associated with long-term fitness costs (i.e., reproductive output, molt, growth, protection against oxidative damage) (Angelier et al. 2013).

HPA axis activity declined at the transition from the pre-parental to parental stages of breeding and is consistent with previous research in Low- and High-Arctic breeding birds (Holberton and Wingfield 2003; Krause et al. 2015; Meddle et al. 2003; Reneerkens et al. 2002; Walker et al. 2015). However, contrary to our original prediction, we did not find a difference in HPA axis activity between birds at the limits of the range compared to those from the historic range during the parental or molt stages. This may suggest that as arctic birds become parental, regardless of location in the range, HPA axis activity must be dampened in order to ensure parental investment (Walker et al. 2015; Wingfield et al. 1995b). Since corticosterone mediates life history trade-offs, maintenance of elevated HPA axis activity through the parental stage may trigger unwarranted entry into the emergency life history stage. Previous research indicated that elevated corticosterone levels and entry into the emergency life history stage during incubation or the nestling phase were correlated with reproductive failure because resources were allocated away from reproduction towards self-maintenance (Astheimer et al. 1995; Spée et al. 2010; Thierry et al. 2013; Wingfield et al. 1983). During molt, the HPA axis activity is typically suppressed, presumably to facilitate the growth of high-quality feathers (Cornelius et al. 2011; Romero and Wingfield 1999). There was a trend, although not significant, for corticosterone to be reduced during molt at the range limits.

### Conclusion

Some species’ ranges are changing at an unprecedented rate and individuals are moving into areas that may differ in quality, thus exposing them to potentially challenging new conditions. In this study, breeding sites for Gambel’s white-crowned sparrows at the northern edge of their range were significantly colder during the early season compared to southern historic sites. Investigating the basic physiology of these individuals at range limits will enable us to better understand the mechanisms that drive species range shifts and ultimately the colonization of novel habitats. Higher body condition index in birds at the northern limit of their range may suggest that selection at some level has occurred. Enhanced HPA axis activity may be just one of many physiological mechanisms underlying adaptation to the challenges of a sub-optimal habitat at the very edge of a population’s range (Wingfield et al. 2015). Male birds at the range limit compared to the historic range had a higher HPA axis activity during the pre-parental stage but no differences were found during the parental or molt stages. This plasticity in the HPA axis may be a result of individual plasticity or genetic selection, but this is difficult to establish without further research. Future studies should address other physiological mechanisms at work, such as changes in hormone receptor density (Liebl and Martin 2013) and modulation of hormone-binding proteins to investigate whether regulation is occurring at multiple levels (Breuner et al. 2003). Such studies are critical for understanding and predicting the extent of species adaptation in an environment that is changing at an unprecedented rate.

## Electronic supplementary material

Breeding Bird Survey (BBS) data from 1993-2012 indicating total number of Gambel’s white-crowned sparrows observed along 20-mile transect conducted on the James Dalton Highway, Alaska. South Fork Koyukuk is located south of the Brooks Range, Dietrich River is in the Brooks Range, and Roche Mountonee and Sagavanirktok Department of Transportation are both to the North of the Brooks Range in the foothills (Sauer et al. 2014) (TIFF 154 kb)

Mean daily wind speed during the breeding season of white-crowned sparrows at the historic range (*gray*) and range limit (*black*) located along the James Dalton Highway, Alaska. The timing of pre-parental (*PP*), parental (*P*), and molt (*M*) stages are indicated by the* horizontal lines* at the top of each graph. Wind speeds were significantly higher at every stage at the range limit compared to historic range (TIFF 116 kb)
