# Supplementary figures and images for: Breeding on the leading edge of a northward range expansion: differences in morphology and the stress response in the arctic Gambel’s white-crowned sparrow
Source: Oecologia. 2015 Oct 1;180:33–44. doi: 10.1007/s00442-015-3447-7 (PMC4698297; doi:10.1007/s00442-015-3447-7)

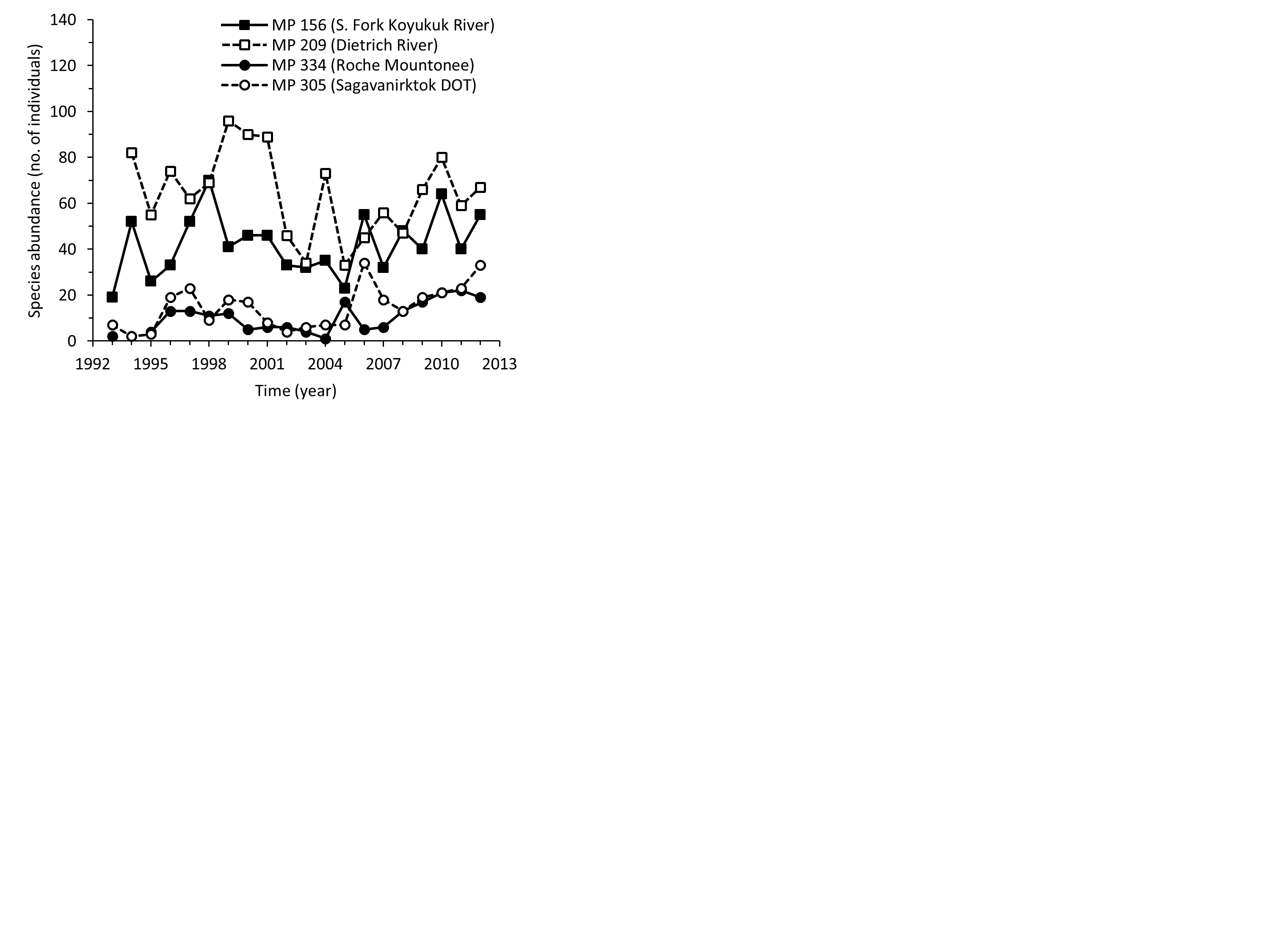

Supplement: Supplementary file 1 — Breeding Bird Survey (BBS) data from 1993-2012 indicating total number of Gambel’s white-crowned sparrows observed along 20-mile transect conducted on the James Dalton Highway, Alaska. South Fork Koyukuk is located south of the Brooks Range, Dietrich River is in the Brooks Range, and Roche Mountonee and Sagavanirktok Department of Transportation are both to the North of the Brooks Range in the foothills (Sauer et al. 2014) (TIFF 154 kb) [file 442_2015_3447_MOESM1_ESM.tif]

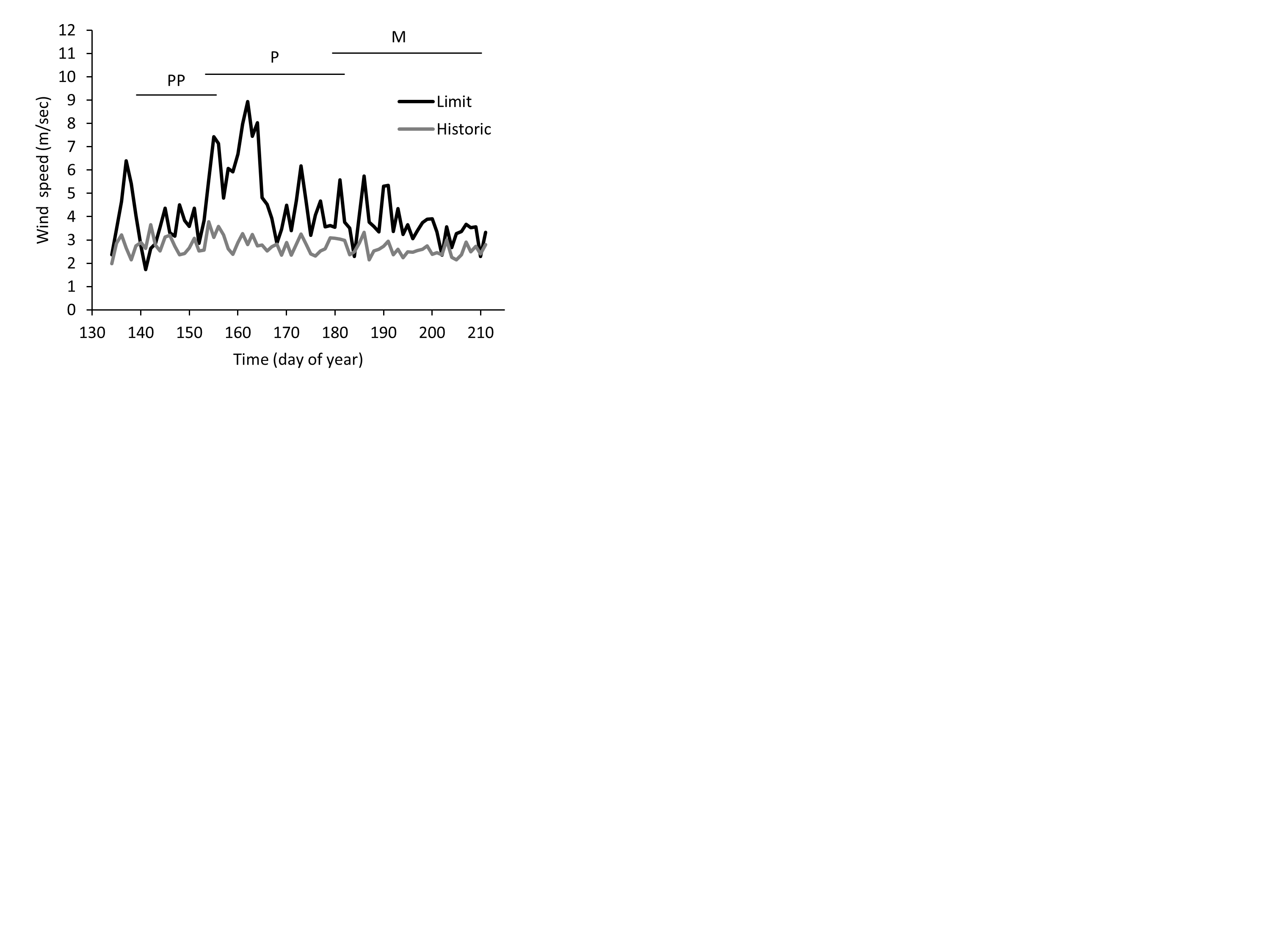

Supplement: Supplementary file 2 — Mean daily wind speed during the breeding season of white-crowned sparrows at the historic range (gray) and range limit (black) located along the James Dalton Highway, Alaska. The timing of pre-parental (PP), parental (P), and molt (M) stages are indicated by the horizontal lines at the top of each graph. Wind speeds were significantly higher at every stage at the range limit compared to historic range (TIFF 116 kb) [file 442_2015_3447_MOESM2_ESM.tif]
